# Pharmacological justification of use of *Solena heterophylla* Lour. in gastrointestinal, respiratory and vascular disorders

**DOI:** 10.1186/s12967-015-0470-8

**Published:** 2015-04-30

**Authors:** Khalid Hussain Janbaz, Tashfeen Akhtar, Fatima Saqib, Imran Imran, Muhammad Zia-Ul-Haq, Chaweeewan Jansakul, Vincenzo De Feo, Marius Moga

**Affiliations:** Faculty of Pharmacy, Bahauddin Zakariya University, Multan, Pakistan; Office of Research, Innovation and Commercialization, Lahore College for Women University, Jail Road, Lahore, 54000 Pakistan; Department of Environmental Sciences, Lahore College for Women University, Jail Road, Lahore, 54000 Pakistan; Faculty of Traditional Thai Medicine, Prince of Songkla University, Hat-Yai, 90112 Thailand; Department of Pharmacy, Salerno University, Fisciano, Italy; Department of Medicine, Transilvania University of Brasov, Brasov, Romania

**Keywords:** *Solena heterophylla* Lour, Spasmolytic activity, Bronchodilator activity, Vasorelaxant activity

## Abstract

**Background:**

*Solena heterophylla* Lour. has traditionally been used in the management of diseases pertaining to gastrointestinal, respiratory and vascular system and present study was undertaken to validate its traditional uses.

**Methods:**

The aqueous ethanolic extract of *Solena heterophylla* Lour (Sh.Cr) was tested *in-vitro* on isolated rabbit jejunum, tracheal and aorta preparations. The responses of tissues were recorded using isotonic transducers coupled with PowerLab data acquisition system.

**Results:**

The aqueous ethanolic extract of *Solena heterophylla* Lour (Sh.Cr) (0.03-1.0 mg/ml) on application to spontaneous contractions in isolated rabbit jejunum preparation exerted relaxant effect through decrease in magnitude and frequency of contractions, caused relaxation of K^+^(80 mM)-induced contractions and shifted the Ca^2+^ concentration response curves toward right in isolated rabbit jejunum preparations in a manner similar to verapamil (a standard Ca^2+^ channel blocker), thus confirming its Ca^2+^ channel blocking activity. The Sh.Cr also caused relaxation of carbachol (1 μM)- and K^+^(80 mM)-induced contractions in isolated rabbit tracheal preparations in a manner comparable to dicyclomine.

**Conclusions:**

The observed relaxant effect may be outcome of anti-muscarinic and Ca^2+^ channel blocking activities. The Sh.Cr (0.03-1.0 mg/ml) against phenyephrine (1 μM)- and K^+^(80 mM)-induced contractions in isolated rabbit aortic preparations exerted a relaxant effect, possibly through Ca^2+^ channel blocking activity. These findings provide a rationale for the folkloric uses of the plant in the management of ailments pertaining to gastrointestinal, respiratory and vascular system.

## Introduction

*Solena heterophylla* Lour. (*Cucurbitaceae*) is a climber plant, distributed widely in India, Pakistan, Afghanistan, Indonesia, Malaysia, Myanmar, Nepal, China, Thailand and Vietnam [1-3]. The plant grows in mixed forests, thickets grasslands, roadsides, and mountain slopes at an altitude of 600–2600 m [4]. The roots are fusiform, about 1.5-2 cm in diameter. The stem and branches are glabrous, petiole are slender, 4–10 mm, puberulent at first but subsequently glabrescent. The leaf blade are variable, ovate, oblong, ovate-triangular, or hastate, undivided or 3-5-lobed, leathery; lobes oblong-lanceolate, lanceolate, or triangular, 8–12 × 1–5 cm^2^, abaxially densely setose or almost glabrous, adaxially densely setose or scabrous, base cordate, margin entire or dentate, apex obtuse or acuminate. Tendrils are slender. The male flowers are umbellate or sub-umbellate; peduncle very short, apically 10-20-flowered; pedicels 2–8 mm; calyx tube 3–5 mm, 3 mm in diameter; segments subulate, 0.2-0.4 mm; corolla yellow or yellow-white; segments triangular, 1–1.5 mm, apex obtuse or acute; filaments filiform, about 3 mm; anther cells curved or conduplicate, puberulent. The female flowers are usually solitary; pedicel 2–10 mm, puberulent; female calyx and corolla are similar to male flowers; ovary ovoid, 2.5-3.5 × 2–3 mm^2^; stigmas 3. The fruits are red-brown, broadly ovoid, oblong, or sub-globose, 2–6 × 2–5 cm^2^. The seeds are gray-white or gray-brown, sub-orbicular or obovate, 5–7 × 5–6.5 mm^2^, smooth or slightly tuberculate. Flowering occurs in May-August and fruiting in June-November [5].

Various parts of this plant possess anti-malarial, anti-diabetic, analgesic, sedative and purgative properties and used to treat toothache, rheumatism and respiratory disorders [6,7]. It is believed to possess invigorating and stimulant properties. The fruits have traditionally been used in the management of common cold, child pneumonia, throat pain and fever; the leaves are applied over inflamed skin, whereas root juice has been used to treat dysuria and spermatorrhoea [4,8]. Phytochemical investigations revealed the presence of behemic acid, columbin and lignoceric acid as plant constituents [4,9]. Scientific investigations on plant extract revealed its hepatoprotective potential, while coumarin and flavonoids isolated from plant were found to inhibit platelet aggregation [10-12]. Moreover, recent study has reported *in-vitro* and *in-vivo* antioxidant activity of methanolic extract of *Solena* whole plant [13] as well as methanolic extract of leaf and stem of *S. heterophylla* [14]. *S. heterophylla* has traditionally been used for the management of gastrointestinal, respiratory and cardiovascular ailments [15,16], but no study exists on validation of these activities. As part of series of experiments in our laboratory on validating tagged biological and physiological activities of medicinal plants [17-22], the current study was designed to investigate and validate the therapeutic potential of *S. heterophylla* in cardiovascular, respiratory and gastrointestinal ailments.

## Materials and methods

### Plant material

The aerial parts of *Solena heterophylla* were collected from hilly areas of Nathia Gali (Abbottabad), Pakistan in August 2011. The plant was authenticated by the expert taxonomist Prof. Dr. Altaf Ahmad Dasti, Institute of Pure and Applied Biology, Bahauddin Zakariya University, Multan, Pakistan and a voucher specimen in preserved in the same University. The plant material was rendered free of foreign contamination by manual picking and allowed to be dried in shade. The dried herbal material was grinded into coarse powder by means of a herbal grinder and was subjected to maceration by soaking (1000 g) in aqeous-ethanolic (70%) mixture in amber colour glass container at room temperature for 7 days with occasional shaking. The material was passed through a muslin cloth and fluid obtained was filtered through Whatmann-1 filter paper. The filtrate was evaporated on a rotary evaporator (Büchi R-200 Switzerland) attached with a vacuum pump (Büchi Vac V-500) and re-circulating chiller (B-740) at 37°C under reduced pressure to a thick dark green paste of semi solid consistency, with an approximate yield of 23%. The extract was stored in air tight jar and all the dilutions were made fresh on the day of experiment [23,24].

### Chemicals

Acetylcholine chloride, carbachol, potassium chloride, verapamil hydrochloride, phenylephrine, magnesium chloride, ethylene tetra-acetic acid (EDTA) were purchased from Sigma Chemicals Co. (St Louis, MO, USA). Calcium chloride, glucose, magnesium sulphate, potassium dihydrogen phosphate, sodium bicarbonate, sodium dihydrogen phosphate and methanol were obtained from Merck (Darmstadt, Germany). Ammonium hydroxide, sodium chloride, and sodium hydroxide were purchased from BDH Laboratory supplies (Poole, England). The chemicals used in these experiments were of highest purity and reagent analytical grade. Stock solutions and subsequent dilutions were made in distilled water on the day of experiment. The drugs were made soluble in vehicles which were without any effect on tissues in control experiments.

### Experimental animals and housing conditions

Animals (♂/♀) used in this study were local strain rabbits (1.0-1.8 kg). These were housed under controlled environmental condition (23–25°C) at the animal house of Faculty of Pharmacy, Bahauddin Zakariya University, Multan. The animals were provided with standard food and tap water *ad libitum.* The animals were deprived of food 24 hr prior to the experiments but were given free access to water. Rabbits were sacrificed following a blow on back of head to be used for *in vitro* studies. All the experiments performed complied with the rulings of Institute of Laboratory Animal Resources, Commission on Life Sciences [25]. The experimental protocols regarding current study were submitted to and approved by the ethical committee meeting held on 16-02-2011 via Notification Number EC/04/2011 dated of the Department of Pharmacy, Bahauddin Zakariya University, Multan.

### *In vitro* experiments

The experiments on isolated tissues were performed by procedures previously described [17-21]. Briefly, we used freshly prepared jejunum, tracheal and aortic tissue segments from the rabbit and maintained adequately in the respective buffer solutions. The detailed elaboration of each tissue extraction procedure is described below under the respective heading of tissue of interest.

### Isolated rabbit jejunum preparations

The crude ethanolic extract of *S. heterophylla* (Sh.Cr) was tested for the possible presence of either spasmolytic or spasmogenic activity by using isolated rabbit jejunum preparations. Isolated rabbit jejunum segments of approximately 2 cm in length were suspended in isolated tissue baths containing Tyrode’s solution, at 37°C, aerated with carbogen (95% O_2_ and 5% CO_2_). The composition of the Tyrode’s solution (mM) was: KCl (2.68), NaCl (136.9), MgCl_2_ (1.05), NaHCO_3_ (11.90), NaH_2_PO_4_ (0.42), CaCl_2_ (1.8) and glucose (5.55). A preload of 1 gm was applied and intestinal responses were recorded through an isotonic transducer by Power Lab Data Acquisition System (AD Instruments, Sydney, Australia) attached to a computer installed with a Lab Chart Software (Version 6). The tissues were allowed to equilibrate for at least 30 min prior to the addition of any drug. Isolated rabbit jejunum preparations exhibit spontaneous rhythmic contractions and allow testing of the antispasmodic (relaxant) effect without application of an agonist [26]. The observed response of the test material was quantified by the application of doses in a cumulative fashion. The relaxant effects on the part of the test substances were taken as the percent change in spontaneous contractions of the preparation recorded immediately before the addition of test substances.

The possible mechanism of the relaxant activity of the test materials were investigated through the relaxation of the observed sustained spasmodic contractions following the exposure to high concentration of K^+^(80 mM) [27]. The test materials were applied in a cumulative manner to the sustained contractions to achieve concentration-dependent inhibitory responses [28]. The observed relaxant effect of the test materials on K^+^ (80 mM)-induced contraction was expressed as percent of the control contractile response.

Calcium channel blocking effect of the test substances were confirmed by the method described by Gilani *et al.* [26]. The isolated rabbit jejunum preparations were allowed to stabilize in normal Tyrode’s solution, which were subsequently replaced for 30 min with Ca^2+^-free Tyrode’s solution to which EDTA (0.1 mM) was added in order to remove calcium from the tissues. This bath solution was further replaced with K^+^-rich and Ca^2+^-free Tyrode’s solution, having the following composition (mM): KCl (50), NaCl (91.04), MgCl_2_ (1.05), NaHCO_3_ (11.90), NaH_2_PO_4_ (0.42), glucose (5.55) and EDTA (0.1). Subsequent to an incubation period of 30 min., cumulative Ca^2+^ concentrations were applied to the tissue bath to obtain control calcium dose–response curves (DRCs). On achievement of the super-imposable control calcium dose–response curves (usually after two cycles), the tissues were then washed and allowed to equilibrated with the plant extract for 1 hr and then the concentration response curves of Ca^2+^ were recorded and compared to the control curves. The DRCs of Ca^2+^ were recorded in the presence of different concentrations of the plant extracts in tissue bath.

### Isolated rabbit tracheal preparations

The rabbit tracheas were dissected out and kept in Krebs solution with the following composition (mM): NaCl (118.2), NaHCO_3_ (25.0), CaCl_2_ (2.5), KCl (4.7), KH_2_PO_4_ (1.3), MgSO_4_ (1.2) and glucose (11.7). The trachea was cleaned free from the surrounding fatty tissues and rings of 2–3 mm width containing 2–3 cartilages were prepared. Each ring was opened by a longitudinal incision on the ventral side opposite to the smooth muscles layer to form a strip with smooth muscles layer in middle and cartilages on both sides. These tracheal preparations were mounted in 20 ml organ bath containing Krebs solution being maintained at 37°C and aerated with carbogen. A preload tension of 1 g was applied and tissue preparations were allowed to be equilibrated for 1 hour prior to any challenge by the drug. Tissue preparations were stabilized by repeated applications of carbachol (1 μM) until constant responses were recorded. The carbachol (1 μM)- and high K^+^(80 mM)-induced sustained contractions were subsequently used for testing of different doses of the test material in a cumulative fashions. The isometric responses were recorded through a Power Lab Data Acquisition System (AD Instruments, Sydney, Australia) attached to a computer installed with a Lab Chart Software (Version 6). The standard drug with Ca^2+^ channel blocking effect (verapamil) was tested on high K^+^(80 mM)- and carbachol- induced spastic contractions in order to confirm the possible mechanism of action.

### Isolated rabbit aorta preparation

The effect of Sh.Cr on systemic vascular resistance was assessed on isolated rabbit aorta preparations. Rabbits of either sex were sacrificed by a blow on the back of head and descending thoracic aorta was dissected out and kept in the normal Krebs solution having composition as above described. It was then cut vertically in 2–3 mm width segments. Each isolated tissue segment was then hung in a tissue organ bath (Radnoti) containing Kreb’s solution aerated with carbogen (95% oxygen and 5% carbon dioxide) at 37°C. A pre-load of 2 g was applied to each preparation and allowed to equilibrate for a period of 1 hr. After equilibration, tissue was stabilized by repeated exposure to K^+^ (80 mM) or phenylephrine (1 μM) depending upon the protocol of the experiment. The vasorelaxant/vasoconstrictive effects of the test substances were studied by addition in tissue organ baths containing pre-stabilized tissue in a cumulative manner. Changes in isometric tension of aortic rings were obtained via fa orce-displacement transducer (Model FORT100, WPI, USA) coupled to a Power Lab data acquisition system (AD Instruments, Sydney, Australia) and a computer running Lab Chart software (version 6).

### Statistical analysis

Data are expressed as mean ± S.E.M. (n = 5 of individual experiments) and median effective concentration (EC_50_) are given with 95% confidence intervals (CI) and the logarithmic dose response curves of different treatments were then plotted using the Computer software “Graphpad Prism” (Graph Pad Software, San Diego, CA, USA).

## Results

### Effect of Sh.Cr on isolated rabbit jejunum preparations

**Figure 1:**
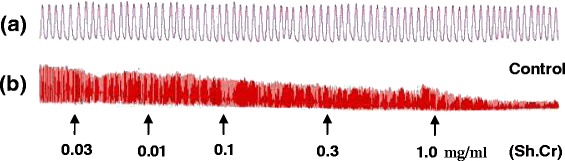
Tracings showing **(a)** the spontaneous contraction of isolated rabbit jejunum and **(b)** the spasmolytic effect of the crude ethanol extract of *Solena heterophylla* (Sh.Cr). Plant extract was added in cumulative manner and values listed were the final tissue bath concentrations, (n = 5).

**Figure 2:**
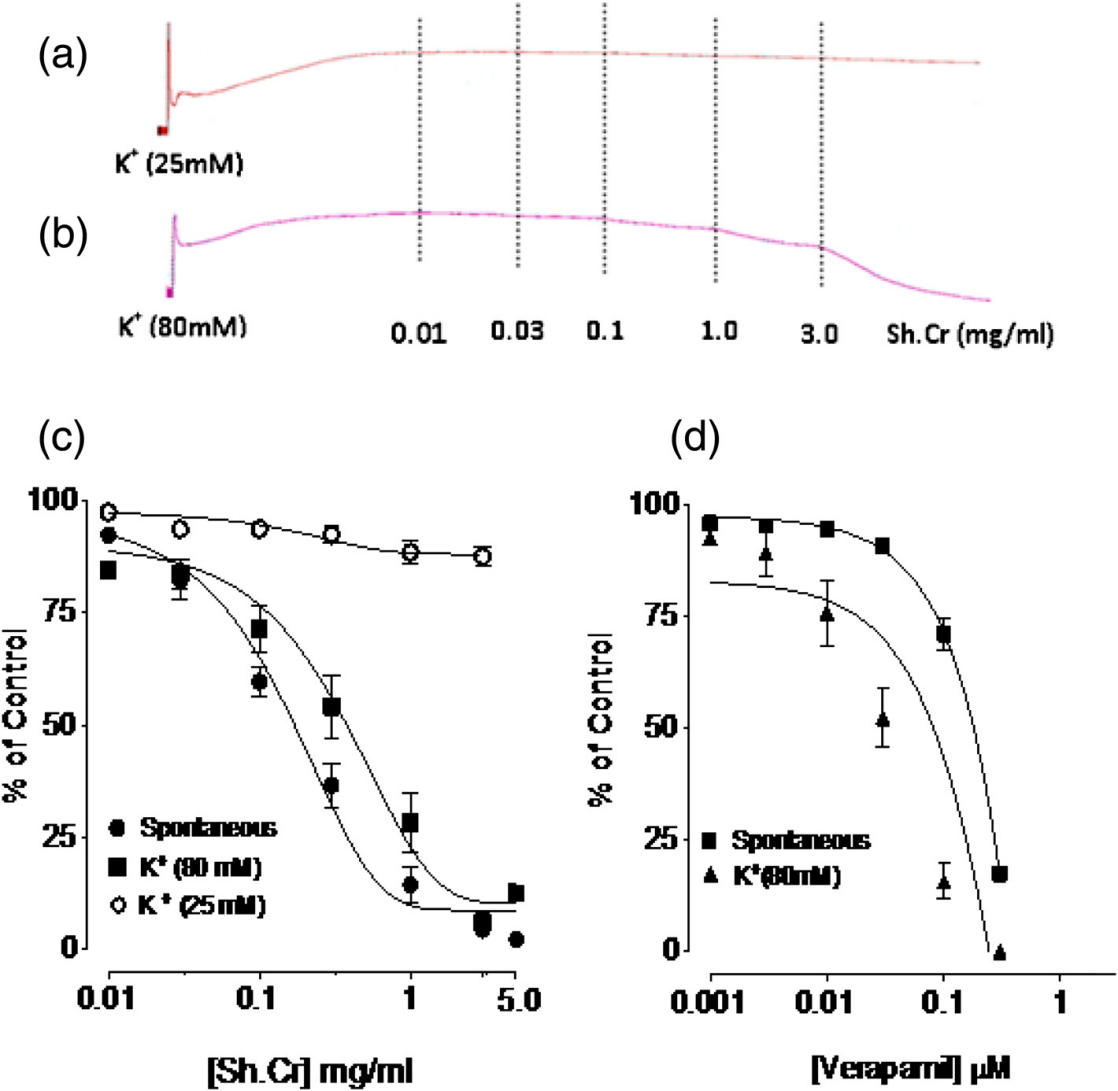
Concentration dependent effect of **(a,b,c)** the ethanol extract of *Solena heterophylla* (Sh.Cr) and **(d)** verapamil on spontaneous, low K^+^ (25 mM) and high K ^+^ (80 mM)-induced contractions in isolated rabbit jejunum preparations. Values are the mean ± SEM, n = 5.

**Figure 3:**
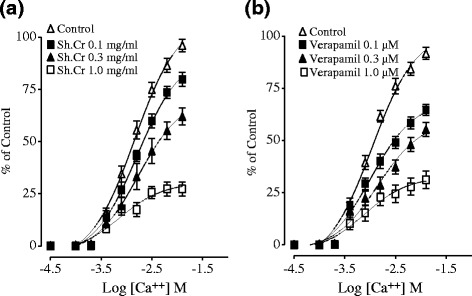
Effect of **(a)** the ethanol extract of *Solena heterophylla* Lour (Sh.Cr) and **(b)** verapamil on concentration response curves of Ca^2+^ in isolated rabbit jejunum preparations. Values are the mean ± SEM, n = 5.

The aqueous ethanolic extract of *Solena heterophylla* Sh.Cr when applied to spontaneous contractions in isolated rabbit jejunum preparations, exerted a relaxant effect in tissue bath concentration-dependent manner, in concentration range of 0.03-1.0 mg/ml, with an EC_50_ value of 0.07002 mg/ml (95% CI: 0.08482-0.1644 mg/ml, n = 5) (Figures 1 and 2). The application of Sh.Cr to K^+^ (25 mM)-induced spastic contractions in isolated rabbit jejunum preparations, resulted in minor relaxant response with an EC_50_ value of 0.3226 mg/ml (95% CI: 0.01782-0.3679 mg/ml, n = 5) (Figure 2a and c), whereas the application to K^+^ (80 mM)-induced spastic contractions caused a complete relaxation, with an EC_50_ value of 0.06824 mg/ml (95% CI: 0.1459-0.2781, n = 5) (Figure 2b and c). Similarly, verapamil also relaxed the spontaneous and K^+^ (80 mM)-induced contractions in isolated rabbit jejunum preparations, with respective EC_50_ values of 0.795 μM (95% C.I: 0.588- 1.105 μM; n = 5) and 0.4511 μM (95% C.I: 0.2944-0.6787 μM; n = 5) (Figure 2d). Moreover, the pretreatment of isolated rabbit jejunum preparations with Sh.Cr (0.1-1.0 mg/ml) caused a rightward shift of Ca^2+^ concentration response curves in a manner similar to that produced by verapamil (Figure 3).

### Effect of Sh.Cr on isolated rabbit tracheal preparations

**Figure 4:**
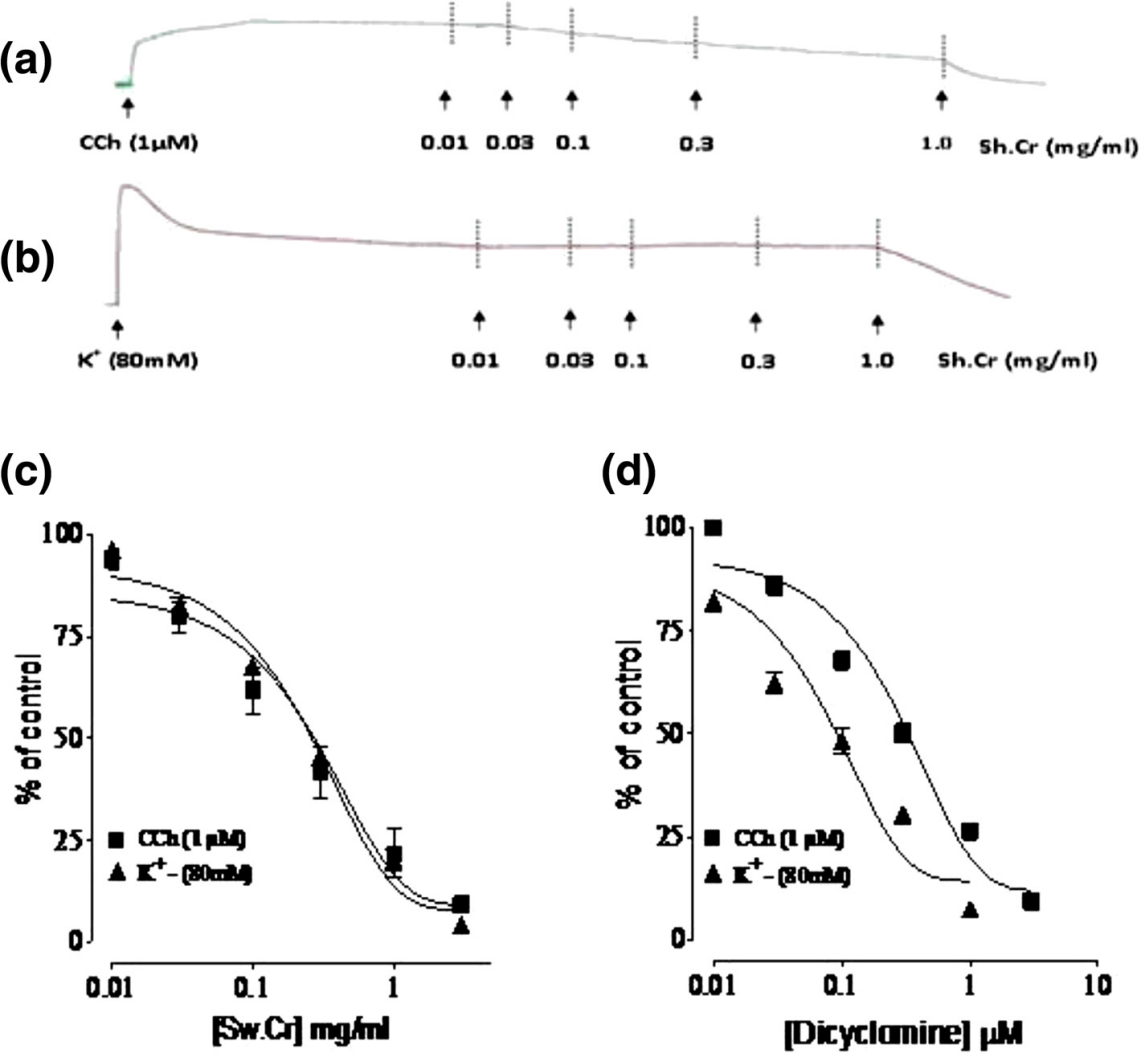
Concentration dependent inhibitory effect of **(a, b and c)** the ethanol extract of *Solena heterophylla* (Sh.Cr) and **(d)** dicyclomine on carbachol (1 μM)- and high K^+^ (80 mM)- induced contractions in isolated rabbit tracheal preparations. Values are the mean ± SEM, n = 5.

The application of Sh.Cr on isolated rabbit tracheal preparations did not produce any response (not shown), however, it exerted a relaxant effect on the carbachol (CCh; 1 μM) and K^+^ (80 mM) induced contractions (Figure 4a,b and c) with respective EC_50_ values of 0.06550 mg/ml (95% CI: 0.09267- 0.1721 mg/ml, n = 5) and 0.06926 mg/ml (95% CI: 0.1422-0.2761 mg/ml, n = 5). The comparison of the above mentioned values shows that EC_50_ of Sh.Cr for CCh-induced contractions is numerically minor if compared to EC_50_ value of Sh.Cr for K^+^ (80 mM)-induced contractions in isolated rabbit tracheal preparations. For this reason, it is possible that some components of Sh.Cr exerted their relaxant effect through the blockade of muscarinic receptors, whereas remaining components may contribute to the relaxant effect through th eblockade of Ca^2+^ channels in a manner comparable to dicyclomine, which caused relaxation of CCh and K^+^ (80 mM) –induced contractions with EC_50_ values of 0.08764 μM (95% CI: 0.05573-0.1317; n = 5) and 0.08846 μM (95% CI: 0.04268-0.09845; n = 5), respectively (Figure 4).

### Effect of Sh.Cr on isolated rabbit aorta preparations

**Figure 5:**
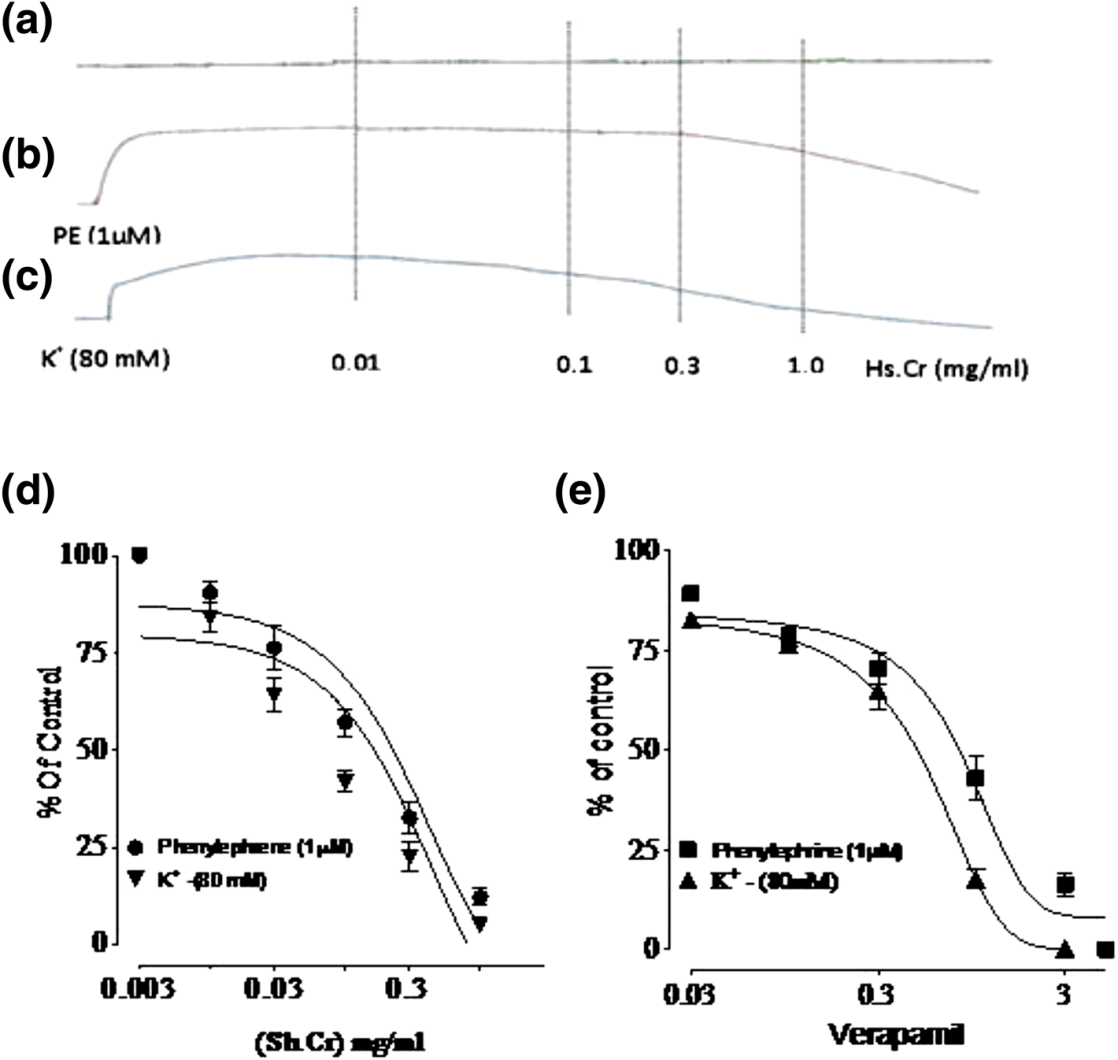
Effect of Sh.Cr on isolated rabbit aorta preparations. **(a)** Unstimulated aortic tissue as control. Concentration dependant inhibitory effect of **(b,c and d)** ethanol extract of *Solena heterophylla* (Sh.Cr) and **(e)** verapamil on phenylephrine (1 μM)- and high K + (80 mM)- induced contractions in isolated rabbit aorta preparations. Values are the mean ± SEM, n = 5.

Sh.Cr did not exert any effect on isolated rabbit aorta preparation in isolated tissue bath concentration range of 0.01-5 mg/ml (Figure 5a). However, it relaxed phenylephrine (1 μM) and K^+^ (80 mM)-induced contractions with respective EC_50_ values of 0.05571 mg/ml (95% CI: 0.04482-0.07642 mg/ml, n = 5) and 0.04955 mg/ml (95% CI: 0.09620-0.1537, n = 5) (Figure 5b, c and d). As the EC_50_ value of Sh.Cr for phenylephrine-induced contractions was found to be high in comparison with the EC_50_ value of Sh.Cr for K^+^(80 mM)-induced contractions, it is possible that the relaxant effect of Sh.Cr on phenylephrine- and K^+^(80 mM)-induced contractions may be mediated through the blockade of Ca^2+^ channels in a manner similar to verapamil, exerting ra elaxant effect on phenyleprine- and K^+^(80 mM)-induced contractions with respective EC_50_ values of of 0.87 μM (95% CI:0.037-5.62; n = 5) and 0.47 μM (95% CI: 0.033-2.11; n = 5) (Figure 5e).

## Discussion

Cardiovascular, respiratory and gastrointestinal problems are some of most common ailments that people face globally in both developing and developed countries. Botanical therapies are still considered as safe by indigenous communities to treat such ailments in developing countries. The current study is aimed to rationalize the folk use of a medicinal plant to cure these ailments. The spasmolytic properties of Sh.Cr were evaluated by its application to spontaneous contractions of isolated rabbit jejunum preparation because these spontaneous rhythmic contractions are suitable for direct testing of relaxant activity without using any agonist [21]. The Sh.Cr exhibited relaxant activity on rhythmic contractions in isolated rabbit jejunum preparations, thus demonstrating its antispasmodic potential.

The contractile activities in smooth muscle preparations are function of increase/decrease in free Ca^2+^ concentration within cytoplasm [29] and the cellular free Ca^2+^ concentration is increased by influx on either via voltage dependent Ca^2+^ channels (VDCs) or release of Ca^2+^ from sarcoplasmic stores [30]. Morevoer, the spontaneous rhythmic contractions in isolated rabbit jejunum preparations are outcome of periodic depolarization subsequent to repolarization, permitting a rapid influx of Ca^2+^ through VDCs at the peak of depolarization [31]. The observed spasmolytic effect of Sh.Cr is likely to be attributed to a decrease in cytoplasmic Ca^2+^ due to the blockade of VDCs or opening of K^+^ channels. The sustained contractions in isolated rabbit jejunum preparations subsequent to K^+^(25 mM) exposure were not relaxed on treatment with Sh.Cr, indicating that the spasmolytic activity of Sh.Cr was independent of K^+^-channels. However, K^+^(80 mM)-induced contractions in isolated rabbit jejunum were found to be relaxed following the treatment with Sh.Cr, indicating that the spasmolytic effect may due to the blockade of rapid influx of extracellular Ca^2+^ through opened VDCs [24,32]. These findings agree with previous studies on medicinal plants [17,33]. These considerations were further confirmed as the previous treatment of isolated rabbit jejunum preparation with Sh.Cr caused decrease in contractile response to Ca^2+^ and rightward shifting of the concentration response curves for Ca^2+^ in a manner similar to verapamil, a standard Ca^2+^ channel blocker [34]. The Ca^2+^ channel blockers are an established class of therapeutic agents and are known to be effective in hyperactive diseases of the gut [35].

The Sh.Cr caused relaxation of carbachol (1 μM)- and K^+^(80 mM)-induced contractions in isolated rabbit tracheal preparations in a manner comparable to dicyclomine and is mediated possibly through antagonism of muscarinic receptors as well as the blockade of Ca^2+^ channels. The anti-muscarinic agents as well as Ca^2+^ channel blockers are useful bronchodilator in conditions of increased sensitivity of the airway [30,31,36,37]. This study provided a scientific basis to validate the traditional uses of the plant in the management of respiratory disorders including asthma, cough, and bronchitis.

The Sh.Cr exerted relaxant effect on phenylephrine (1 μM)- and K^+^(80 mM)-induced contractions in isolated rabbit aorta preparations, but phenylephrine-induced contractions were relaxed at increased tissue bath concentrations, indicating that the observed relaxant effect may possibly be mediated through the blockade of voltage dependent Ca^2+^ channel [32,38]. The relaxant effect on isolated rabbit aorta preparations may provide a scientific basis to validate the use of *Solena heterophylla* in the management of hypertension.

## Conclusions

The observed relaxant effect may be outcome of anti-muscarinic and Ca^2+^ channel blocking activities. The *Solena heterophylla* exerted a relaxant effect against phenyephrine (1 μM)- and K^+^(80 mM)-induced contractions in isolated rabbit aortic preparations, possibly through Ca^2+^ channel blocking activity. These findings provide a rationale for the folkloric uses of the plant in the management of ailments pertaining to gastrointestinal, respiratory and vascular system. However, more detailed studies are required to establish the safety, efficacy and toxicity of this plant and to isolate the bioactive constituents.
